# Novel anti-*Acanthamoeba* effects elicited by a repurposed poly (ADP-ribose) polymerase inhibitor AZ9482

**DOI:** 10.3389/fcimb.2024.1414135

**Published:** 2024-05-28

**Authors:** Lijun Chen, Wei Han, Wenwen Jing, Meng Feng, Qingtong Zhou, Xunjia Cheng

**Affiliations:** ^1^ Department of Medical Microbiology and Parasitology, School of Basic Medical Sciences, Fudan University, Shanghai, China; ^2^ Research Center for Intelligent Computing Platforms, Zhejiang Lab, Hangzhou, China; ^3^ Department of Pharmacology, School of Basic Medical Sciences, Fudan University, Shanghai, China

**Keywords:** *Acanthamoeba*, poly (ADP-ribose) polymerase, transcriptomics, DNA damage, drug

## Abstract

**Introduction:**

Acanthamoeba infection is a serious public health concern, necessitating the development of effective and safe anti-Acanthamoeba chemotherapies. Poly (ADP-ribose) polymerases (PARPs) govern a colossal amount of biological processes, such as DNA damage repair, protein degradation and apoptosis. Multiple PARP-targeted compounds have been approved for cancer treatment. However, repurposing of PARP inhibitors to treat Acanthamoeba is poorly understood.

**Methods:**

In the present study, we attempted to fill these knowledge gaps by performing anti-Acanthamoeba efficacy assays, cell biology experiments, bioinformatics, and transcriptomic analyses.

**Results:**

Using a homology model of Acanthamoeba poly (ADP-ribose) polymerases (PARPs), molecular docking of approved drugs revealed three potential inhibitory compounds: olaparib, venadaparib and AZ9482. In particular, venadaparib exhibited superior docking scores (−13.71) and favorable predicted binding free energy (−89.28 kcal/mol), followed by AZ9482, which showed a docking score of −13.20 and a binding free energy of −92.13 kcal/mol. Notably, the positively charged cyclopropylamine in venadaparib established a salt bridge (through E535) and a hydrogen bond (via N531) within the binding pocket. For comparison, AZ9482 was well stacked by the surrounding aromatic residues including H625, Y652, Y659 and Y670. In an assessment of trophozoites viability, AZ9482 exhibited a dose-and time-dependent anti-trophozoite effect by suppressing Acanthamoeba PARP activity, unlike olaparib and venadaparib. An Annexin V-fluorescein isothiocyanate/propidium iodide apoptosis assay revealed AZ9482 induced trophozoite necrotic cell death rather than apoptosis. Transcriptomics analyses conducted on Acanthamoeba trophozoites treated with AZ9482 demonstrated an atlas of differentially regulated proteins and genes, and found that AZ9482 rapidly upregulates a multitude of DNA damage repair pathways in trophozoites, and intriguingly downregulates several virulent genes. Analyzing gene expression related to DNA damage repair pathway and the rate of apurinic/apyrimidinic (AP) sites indicated DNA damage efficacy and repair modulation in Acanthamoeba trophozoites following AZ9482 treatment.

**Discussion:**

Collectively, these findings highlight AZ9482, as a structurally unique PARP inhibitor, provides a promising prototype for advancing anti-Acanthamoeba drug research.

## 1 Introduction

*Acanthamoeba* species, are free-living microorganisms widely distributed in soil, water, and vegetation around the world (Andalib et al., 2022; Zhou et al., 2022), posing a serious public health concern. Outbreaks of amoebic keratitis have afflicted developed countries, such as New Zealand, USA, and UK (Carnt et al., 2018; McKelvie et al., 2018; Scruggs et al., 2019), as well as developing countries in various public water sources (Masangkay et al., 2022; Elseadawy et al., 2023). *Acanthamoeba* spp. cause sight-threatening keratitis (Yee et al., 2021; Maier et al., 2022; Carnt et al., 2023), epithelial disorders, and fatal granulomatous amoebic encephalitis (GAE), particularly in immunocompromised individuals (Martinez and Visvesvara, 1997; Cope et al., 2016; Zhang and Cheng, 2021). GAE, a rare yet usually fatal central nervous system infection resulting from *Acanthamoeba* spp., predominantly affects chronically ill individuals, yielding over 90% mortality (Kot et al., 2021). *Acanthamoeba* keratitis (AK), jeopardizes vision, and poses diagnostic and treatment challenges. Treating AK is prolonged and demanding, although trophozoites, and immature cysts are substantially more responsive to multiple therapies than mature cysts. These facts underscore the pressing need for efficacious treatments against this parasitic pathogen.

Treatment of this neglected pathogen is hampered by two major challenges: adverse effects and drug resistance. Current therapy involves polyhexamethylene biguanide (PHMB; 0.02%-0.08%) and chlorhexidine (0.02%), both of which damage the cornea and cause corneal epitheliopathy (Shing et al., 2021). Miltefosine, an oral alkylphosphocholine for treating amoebas and leishmaniasis, exhibits *in vitro* efficacy against *Acanthamoeba* species (Schuster et al., 2006) as well as clinical success (Avdagic et al., 2021; Thulasi et al., 2021). However, the resistance and toxicity of miltefosine are frequently reported (Matoba et al., 2021; Thulasi et al., 2021). The capacity of *Acanthamoeba* to endure harsh environments and treatment therapy without genetic resistance, but rather by transiently halting growth, slowing metabolism, and metabolically transitioning to dormant cysts, exacerbates treatment challenges. Consequently, current treatment regimens often exhibit limitations, highlighting the urgent need to identify safe and effective therapeutic agents against *Acanthamoeba* spp., optimizing treatment while minimizing adverse effects.

Drug repurposing, utilizing existing therapeutic agents for novel indications (Ashburn and Thor, 2004; Miró-Canturri et al., 2019), offers a promising approach. Computational methods, encompassing genetic association, molecular docking, and signature matching, play crucial roles in this strategy (Pushpakom et al., 2019). Poly (ADP-ribose) polymerases (PARPs) modify target proteins with multiple ADP-ribose units, governing vital cellular, and biological processes, such as DNA damage repair, protein degradation, apoptosis, necrosis, stress responses, and immune function (Perina et al., 2014). Phylogenetic analysis of PARP catalytic domains has revealed six clades (Citarelli et al., 2010), with the human genome harboring 17 PARPs in five distinct clades. PARP inhibition is linked to DNA damage and subsequent apoptosis (Ciccia and Elledge, 2010), which is advantageous for cancer treatment. Of note, the persistent efforts from pharmacological companies have brought four approval PARP inhibitors (olaparib, talazoparib, rucaparib and niraparib)for the treatment of ovarian, fallopian tube, breast, prostate and peritoneal cancers (Curtin and Szabo, 2020). Common side effects of clinically evaluated PARP inhibitors are fatigue, gastrointestinal effects (including nausea/vomiting, abdominal pain and diarrhea) and thrombocytopenia, which are generally mild (grade 1 or 2) but could be stronger in rare cases. PARPs’ involvement in cell death in protozoan parasites exhibiting heightened damage has been extensively investigated (Fernández Villamil and Vilchez Larrea, 2020), with one study examining the use of PARP inhibitors against *Trypanosoma cruzi* infection (Vilchez Larrea et al., 2012). These observations highlight the feasibility and advantage of utilizing PARP inhibition for the development of safe and effective anti-*Acanthamoeba* therapeutic agents. Nonetheless, to the best of our knowledge (Siddiqui et al., 2017), data on PARP sequences, classifications, regulatory roles, inhibitory impact, and proteomic response in *Acanthamoeb*a spp. remain scarce.

In the present study, we attempted to fill these knowledge gaps by performing anti-*Acanthamoeba* efficacy assays, cell biology experiments, bioinformatics, and transcriptomic analyses. Our findings confirmed the capability of *Acanthamoeba* spp. to regulate its own growth environment and employ diverse metabolic pathways, providing insights into PARP inhibition and its robustness in *Acanthamoeba* spp.

## 2 Materials and methods

### 2.1 Molecular docking

Molecular docking was performed by Schrödinger Suite 2018–1 (New York, NY, USA). Homology model of *Acanthamoeba castellanii* PARP L8GH34 (**Supplementary Table S1**) was built mainly based on the X-ray structure of the PARP1 catalytic domain in complex with the inhibitor olaparib (PDB code: 7KK4), exhibiting a sequence identity of 59.4%. Protein structure and ligand compound library including the FDA-approved drugs and reported PARP inhibitors (3,158 compounds) were prepared using the Protein Preparation Wizard and LigPrep modules in Schrödinger Suite, employing default settings, respectively. Receptor grid was generated by Receptor Grid Generation tool in Schrödinger Suite while the grid boxes were defined as a 10×10×10 Å^3^ region centered at the inhibitor olaparib. Two levels of molecular docking were performed. First, the prepared 3,158 compounds underwent standard precision (SP) docking, with the top 10% scoring compounds advancing to an extra precision (XP) docking phase, employing a refined scoring function to minimize false positives. Subsequently, XP docking poses with the leading 200 docking scores underwent Prime MM-GBSA computation, where residues within 5 Å of the docked ligands were relaxed using the “Minimize” sampling method. Finally, by visual inspection of the optimized docking pose and considering the XP docking scores, calculated binding free energies (MM-GBSA dG Bind), and chemotype diversity, nine compounds were selected for the subsequent experimental validation (**Table 1**).

**Table 1:** Identification of selected experimental candidates through virtual screening targeting the representative *A. castellanii* PARP (UniProt accession code: L8GH34).

Compound Name	Docking score	MMGBSA dG Bind (kcal/mol)	Pocket residues
Venadaparib	−13.71	−89.28	N531, E535, G626, Y659, S667, Y670
AZ9482	−13.20	−92.13	G626, R641, Y659, S667, Y670
Olaparib	−13.02	−84.68	G626, R641, Y659, S667, Y670
AZD2461	−12.83	−78.23	G626, Y659, S667, Y670
KU0058948	−12.64	−90.88	E535, G626, Y659, S667, Y670
Talazoparib	−10.31	−70.43	K524, H625, G626, S667, Y670
Azilsartan	−9.04	−84.35	G626, R641, A643, Y652, H625, S667, Y670
Bopindolol	−8.68	−56.44	K524, H625, G626, Y652, M653, Y659, Y670, E752
Trovafloxacin	−8.53	−72.75	K524, R641, M653, Y659, Y670

### 2.2 Amoeba cultivation

*Acanthamoeba castellanii* (ATCC 30011 strain) was obtained from the American Type Culture Collection. Following established protocols (Deng et al., 2015), trophozoites were axenically cultured in peptone–yeast–glucose (PYG) medium. This medium comprised 10 g of proteose peptone, 10 g of yeast extract, 10 g of glucose, 5 g of NaCl, 0.95 g of L-cysteine, 3.58 g of Na2HPO4·12H2O, and 0.68 g of KH2PO4 per liter of deionized distilled water. The culture was incubated at 26°C, and trophozoites, harvested during the late log phase after 48 h of subculture, were used for subsequent analyses.

### 2.3 Real time quantitative polymerase chain reaction

Total RNA was isolated using the RNeasy Plus Mini Kit (74134; QIAGEN, Hilden, Germany), and cDNA was synthesized using the PrimeScript™ II 1st Strand cDNA Synthesis Kit (6210A; Takara Bio, China). Reactions were performed in a 96-well plate with SYBR Premix Ex Taq (Takara, Dalian, Liaoning, China) and primers targeting *A. castellanii PARP* (L8GH34, L8H4Q2, L8GH34), *RAD50*, *RAD51* and *MRE11*. Primers for these genes, sourced from published sequences (**Supplementary Table S2**), facilitated real-time quantitative polymerase chain reaction (RT-PCR) on an ABI 7500 Real-Time PCR system (Applied Biosystems, Foster City, CA, USA). The 2^-△△Ct^ method was used to calculate the relative expression of each primer between the control and treatment groups, with values normalized to the 18S rDNA reference housekeeping gene.

### 2.4 Trophozoites viability assay

Obtained from TargetMol (Wellesley Hills, MA, USA), talazoparib, AZD2461, azilsartan, trovafloxacin, olaparib, and venadaparib were dissolved in 100% dimethyl sulfoxide (DMSO; Sigma-Aldrich, St. Louis, MO, USA). AZ9482 was obtained from MCE (MedChem Express, Monmouth Junction, NJ). Although talazoparib, AZ9482, AZD2461, azilsartan, and trovafloxacin were prepared at 10 mM concentrations, Olaparib, venadaparib and AZ9482 were prepared at 200 mM concentrations. *A. castellanii* trophozoites were seeded at the log growth phase (10^4^ trophozoites per well) in a 96-well white microplate (Eppendorf, Hamburg, Germany). Incubation with medium containing varied olaparib, venadaparib and AZ9482 concentrations (100, 200, 300, 400, and 500 μM) was performed for 24 h, 48 h and 72 h at 26°C. Trophozoites viability assay results, derived from at least three wells per condition and compared with diluted DMSO, were evaluated using CellTiter-Glo (Promega, Madison, WI, USA), as per the manufacturer’s guidelines. Subsequently, 100 μL of CellTiter-Glo reagent was introduced per well, mixed for 2 min on an orbital shaker, and incubated at room temperature for 10 min. Luminescence was recorded on the FlexStation^®^ 3 Multi-Mode Microplate Reader (Molecular Devices, MD, USA), with growth curves subsequently generated using GraphPad Prism 8. The morphological changes of trophozoites was observed using the CFSE staining (Dojin Kagaku) following the manufacturer’s protocol. Treated trophozoites (1×10^6^) were incubated with CFSE working solution (50 μM) for 30 min at 26°C. After treatment with CFSE working solution, the trophozoites were wash twice with cold PBS. Live trophozoites suspensions were placed onto glass slides using Cytospin 4 Cytocentrifuge (Thermo Fisher Scientific) and mounted with coverslips. The fluorescence images were obtained using a Nikon ECLIPSE FN1 microscope (λEx = 488 nm and λEm = 516 nm). Images were merged using the ImageJ software (National Institute of Health, Bethesda, MD).

### 2.5 Amoeba apoptosis analysis

*A. castellanii* trophozoites apoptosis was assessed using an Annexin V-fluorescein isothiocyanate (FITC) apoptosis detection kit, following the manufacturer’s instructions (Sigma-Aldrich). *A. castellanii* trophozoites (6 × 10^5^ trophozoites/well) were cultured with various concentrations of AZ9482 for 24 h in six-well plates. Subsequently, trophozoites were detached from the plates, collected in transparent centrifuge tubes (15 mL), and centrifuged at 800 × g for 5 min. These trophozoites underwent two phosphate-buffered saline (PBS) washes and were resuspended in 1× binding buffer. Subsequently, 2.5 μL of propidium iodide (PI) and 1.25 μL of Annexin V-FITC were added per tube (500 μL). The tubes were incubated for 10 min in darkness at room temperature. Fluorescence-activated trophozoite sorting was performed using a FACSAria instrument (BD Biosciences, San Jose, CA, USA) with a 488-nanometer argon excitation laser, as previously described (Deng et al., 2015). Analysis gates were defined using untreated amoebae, and FlowJo 10.8.1 software (FlowJo LLC, Ashland, OR, USA) was used for data analysis.

### 2.6 DNA damage determination

Genomic DNA was extracted using the DNeasy Blood & Tissue Kit (Catalog No. 69506; QIAGEN), following the manufacturer’s protocol. DNA concentrations were quantified using a BioPhotometer^®^ D30 (Eppendorf). Trophozoite DNA damage was assessed using the DNA Damage-AP Sites-Assay Kit (AP sites, Colorimetric; Abcam, ab211154; Cambridge, UK). DNA was diluted to 100 μg/mL in TE buffer [10 mM Tris (pH 7.5) and 1 mM EDTA], and 5 μL of purified genomic DNA was mixed with 5 μL of 10 mM Aldehyde Reactive Probe (ARP) solution in a MaxyClear microcentrifuge tube (1.5 mL; Corning, NY, USA) and incubated for 1 h at 37°C. To each sample tube, 90 μL of TE buffer, 1 μL of 10 mg/ml glycogen solution, 10 μL of 3M sodium acetate solution (pH5.5), and 300 μL of absolute ethanol were added and mixed, followed by incubation at −20°C for 30 min and centrifugation at 14,000 × g for 20 min at 4°C. DNA pellets were rinsed three times with 70% ethanol, air-dried for 10 min, and dissolved in 25 μL of TE buffer. DNA concentration was measured using the BioPhotometer^®^ D30. ARP-derived DNA samples were diluted to 1 μg/mL in TE buffer. DNA Binding Solution (50 μL) was then added to the provided DNA high-binding plate well, incubated overnight at room temperature on an orbital shaker, and microwells were washed three times in 250 μL of 1× wash buffer. Subsequently, 100 μL of diluted streptavidin–enzyme conjugate (1:1000 dilution in 1× wash buffer) was added to each well, incubated for 1 h at 37°C, and microwells were washed three times in 250 μL of 1× wash buffer. Following the addition of 100 μL of substrate solution, microwells were incubated at room temperature for 10 min on an orbital shaker. The reaction was halted with 100 μL of stop solution, and absorbance at OD 450 nm was immediately measured using a microplate reader (Model 680, Bio-Rad, CA, USA). Corrected absorbance values for each standard were plotted against AP sites per 105 base pairs, and the number of AP sites in treated samples was compared with that in untreated control samples to quantify DNA damage levels.

### 2.7 RNA-seq experiment and data analysis

Total RNA was isolated from each trophozoites of *A. castellanii* sample by using an RNeasy^®^ Plus Mini kit (Qiagen, Germany). RNA quality was examined by gel electrophoresis and with Qubit (Thermo, Waltham, MA, USA). For RNA sequencing, RNA samples from three biological replicates were separated into three independent pools, each comprised of two or three distinct samples, at equal amounts. Strand-specific libraries were constructed using the TruSeq RNA sample preparation kit (Illumina, San Diego, CA, USA), and sequencing was carried out using the Illumina Novaseq 6000 instrument. The raw data was handled by Skewer and data quality was checked by FastQC v0.11.2 (http://www.bioinformatics.babraham.ac.uk/projects/fastqc/). The read length was 2×150 bp. Clean reads were aligned to *A. castellanii* str. Neff using STAR- StringTie. The expression of the transcript was calculated by FPKM (Fragments Per Kilobase of exon model per Million mapped reads) using Perl. Differentially expression transcripts (DETs) were determined using the MA-plot-based method with Random Sampling (MARS) model in the DEGseq package between different time points (12 hpt vs. 0 hpt, 36 hpt vs. 0 hpt, 72 hpt vs. 0 hpt). Generally, in MARS model, M = log_2_C1 - log_2_C2, and A = (log_2_C1 + log_2_C2)/2 (C1 and C2 denote the counts of reads mapped to a specific gene obtained from two samples). The thresholds for determining DETs are P < 0.05 and absolute fold change ≥ 2. Then DETs were chosen for function and signaling pathway enrichment analysis using GO and KEGG database. The significantly enriched pathways were determined when P <0.05 and at least two affiliated genes were included.

### 2.8 Statistical analysis

All statistical analyses were performed using GraphPad Prism 8 (GraphPad Software, Version 8.0, San Diego, USA). Comparison between control and treatment groups was achieved using one- way ANOVA or the Student’s t-test. Data are presented as means ± standard deviations (SDs), derived from a minimum of three independent experiments for each sample. Significance was defined as p < 0.05 across all analyses.

## 3 Results

### 3.1 PARPs in Acanthamoeba castellanii

The UniProtKB (https://www.uniprot.org/) and NCBI protein (https://www.ncbi.nlm.nih.gov/protein/) databases (Sayers et al., 2022; UniProt Consortium, 2023) were searched, leading to the identification of 28 distinct proteins designated as “poly [ADP-ribose] polymerase” or “poly(ADP-ribose) polymerase” within the organism”*Acanthamoeba castellanii* str. Neff” (**Supplementary Table S1**). Despite varying sequence lengths (135–3016 amino acids), all of these 28 proteins exhibited a PARP catalytic domain within the “Family and Domains” section of UniProtKB, a trait inferred from signature matches (Doğan et al., 2016).

**Figure 1:**
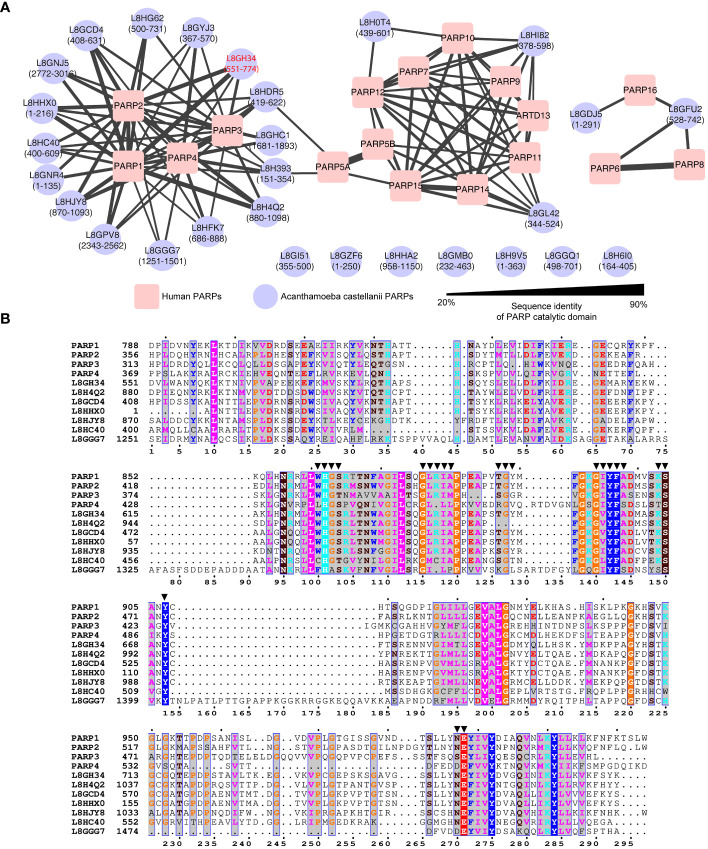
Sequence analysis of *Acanthamoeba castellanii* PARPs. **(A)** Sequence similarity network of *Acanthamoeba castellanii* and *Homo sapiens* PARPs. The *Acanthamoeba castellanii* PARPs were clustered into to the human PARPs based on internal sequence similarities of the catalytic domain. Catalytic domain residues are indicated in parentheses. Network visualized using Cytoscape v.3.10.0, with nodes colored for species identification (red square: *Homo sapiens*; blue circle: *A*. *castellanii*), and edge width reflecting sequence identity. Edges between nodes are included only if the similarity between a pair of sequences was greater than an E-value threshold cutoff of 1E^−15^. One specific isoform (UniProt accession code: L8GH34) exhibited the highest sequence identity relative to either PARP1 (59.4%) or PARP2 (53.1%) has been highlighted in red. **(B)** Sequence alignment of PARP catalytic domain among human PARP1, PARP2, PARP3, PARP4 and seven representative *A*. *castellanii* PARPs. Residues within 4.5 Å of inhibitors reported in human PARP1/PARP2 structures (PDB codes: 3KJD, 4TVJ, 7AAD, and 4UND) are highlighted by solid triangles.

To elucidate the potential classification of these 28 A*. castellanii* PARPs, a sequence similarity network was constructed, incorporating 17 Homo sapiens, and 28 A*. castellanii* PARPs based on the catalytic domain (**Figure 1A**; **Supplementary Figure S1**) (Amé et al., 2004). Among these, 16 A*. castellanii* PARPs were grouped into the human PARPs 1/2/3/4 subfamily (Hottiger et al., 2010), where a specific isoform (UniProt accession code: L8GH34) exhibited the highest sequence identity relative to either PARP1 (59.4%) or PARP2 (53.1%). Five *A. castellanii* PARPs were likely associated with distinct PARP subfamilies (corresponding to PARP10 and PARP16), whereas the remaining seven PARPs remained unclassified owing to pronounced sequence disparities with known human PARPs.

Consequently, our focus centered on the 16 A*. castellanii* PARPs within the human PARPs 1/2/3/4 subfamily, as depicted in the sequence alignments of the PARP catalytic domain (**Figure 1B**). Notably, these representative *A. castellanii* PARPs share the conserved histidine–tyrosine–glutamate (H862–Y896–E988 in PARP1) catalytic triad, designated as the ART signature sequence, which is crucial for PARP activity (Steffen et al., 2013; Barkauskaite et al., 2015). Of note, the H–Y–E motif is exclusively observed in PARPs 1–5, unlike other PARPs, such as 6–8, 10–12, and 14–16, functioning predominantly as mono(ADP-ribosyl) transferases *in vitro*, where glutamate is naturally replaced by leucine, isoleucine, or valine (D’Amours et al., 1999; Barkauskaite et al., 2015). Examination of the ligand-binding pocket revealed a high degree of conservation in pocket residues between human PARPs 1–4 and *A. castellanii* PARPs (**Figure 1B**), supporting the potential feasibility of drug repurposing and structure-based drug screening.

### 3.2 Virtual screening of approved drug agonists against *A. castellanii* PARP

**Figure 2:**
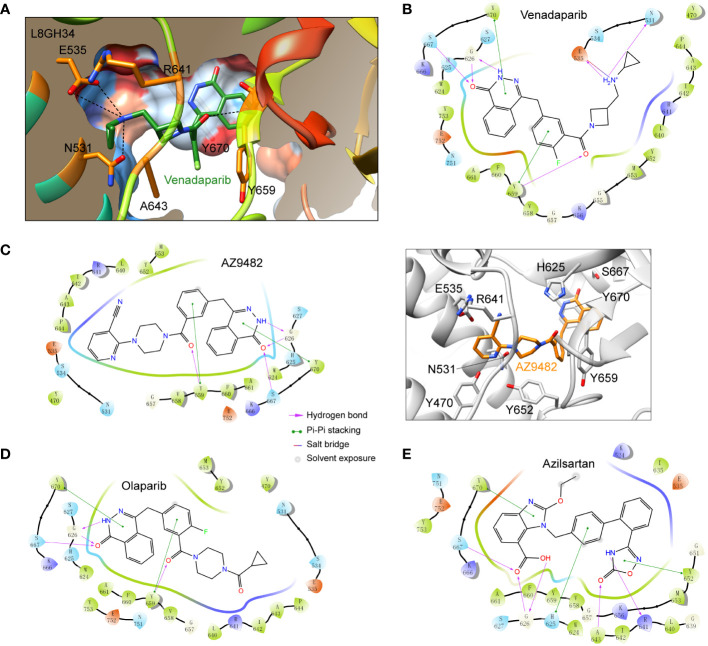
Docking poses of representative approved drugs in the homology model of *A*. *castellanii* PARP (UniProt accession code: L8GH34). **(A)** Surface representation of predicted venadaparib-binding pocket in L8GH34. PARP depicted in surface representation, color-coded from dodger blue (most hydrophilic region) to orange–red (most hydrophobic region). Key residues involved in venadaparib recognition are shown as sticks. Polar interactions are shown as black dashed lines. **(B–D)** Two-dimensional (2D) diagrams of ligand–protein interactions for three representative ligands: venadaparib **(B)**, AZ9482 **(C)**, olaparib **(D)**, and azilsartan **(E)**. PARP residues are highlighted for positive charge (purple), negative charge (pink), polarity (blue), and hydrophobicity (green) of amino acids.Structural representation of the ligand-protein interactions was shown for AZ9482 [**(C)**, right], where residues involved in the AZ9482 recognition are shown as sticks.

To identify potential inhibitors targeting *A. castellanii* PARPs, we first constructed a homology model of a representative *A. castellanii* PARP (UniProt accession code: L8GH34) based on the X-ray structure of the PARP1 catalytic domain in complex with the inhibitor olaparib (PDB code: 7KK4). Subsequently, we performed virtual screening of an approved drug library (Ryan et al., 2021; Zhou et al., 2021), using both rigid docking (Glide SP and XP protocols) and binding free energy estimation. The former was utilized to isolate these docking pose of best docking score, while the latter further relaxed the predicted ligand-PARP1 complex and calculated the binding free energy by Prime MM-GBSA. This approach led us to identify nine potential drug candidates for further validation (**Table 1**; **Supplementary Figure S2**). Among these compounds, venadaparib exhibited the highest docking score (−13.71) and a favorable predicted binding free energy (−89.28 kcal/mol), followed by AZ9482 (−13.20 and −92.13 for docking score and binding free energy, respectively) (**Table 1**). Venadaparib and olaparib share common interactions at one end, involving hydrogen bonds (via G626 and S667) and pi–pi stacking (via Y659 and Y670) while differing in chemotype at the opposite terminus (**Figures 2A, B**, **2D**). The positively charged cyclopropylamine in venadaparib fitted well into the cleft, forming a salt bridge (via E535) and a hydrogen bond (via N531), distinguishing it from olaparib. AZ9482 was stabilized by both stacking interactions from surrounding aromatic residues (such as H625, Y652, Y659 and Y670) and multiple hydrogen bonds with the backbone atoms of Y659 and G626, as well as the sidechain atom of S627 (**Figure 2C**). Additionally, azilsartan adopted a 3-phenyl-4,5-dihydro-1,2,4-oxadiazol-5-one moiety, engaging in multiple interactions, including three hydrogen bonds (via A643 and R641) and a stacking interaction with Y652 (**Figure 2E**).

### 3.3 AZ9482 inhibits *A. castellanii* viability more effectively than others

**Figure 3:**
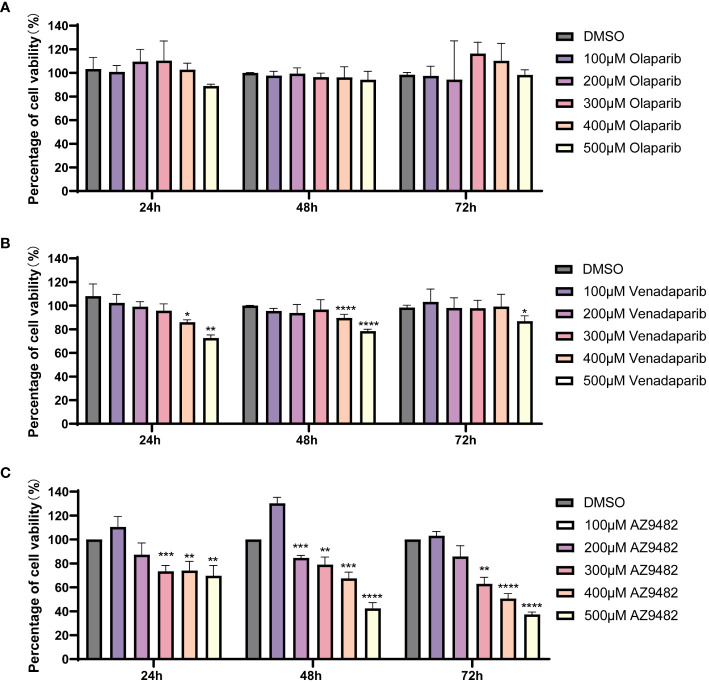
Suppression of *A*. *castellanii* trophozoite viability by PARP inhibitors. **(A, B)** Viability of *A*. *castellanii* trophozoites after venadaparib and olaparib treatment (0–500 μM) for 24, 48, and 72 h determined using the CellTiter-Glo assay. **(C)** Viability of *A*. *castellanii* trophozoites following AZ9482 treatments (0–500 μM) for 24, 48, and 72 h. Data represent means ± SDs from triplicate experiments. DMSO-treated trophozoites were used as the negative control (*p < 0.05; **p < 0.01, *** *p* < 0.001,**** *p* < 0.0001).

To assess the *in vitro* efficacy of the identified drugs, we employed the CellTiter-Glo^®^ Luminescent cell viability assay. Out of the nine hits from virtual screening, seven compounds were obtained, and dissolved in DMSO, whereas two were excluded owing to limited availability (KU0058948 and bopindolol). Four compounds (trovafloxacin, talazoparib, AZD2461, and azilsartan) at 20 μM exhibited no significant inhibition of *A. castellanii* trophozoite viability. These compounds could not be further concentrated for experimentation due to the toxic effects of DMSO (Saunders et al., 1992; Siddiqui et al., 2016). Consequently, olaparib, venadaparib and AZ9482 were selected for further investigations. No significant inhibitory effect was detected on *A. castellanii* trophozoite growth after olaparib treatment (**Figure 3A**). Treatment with 400 and 500 μM venadaparib led to significant reductions in trophozoite viability after 24 h, 48 h and 72 h (*p* < 0.05; **Figure 3B**), respectively, compared with the DMSO-treated group. Specifically, there are exclusively showed a significant time-and dose-dependent decrease in the trophozoites viability, after AZ9482 treatment with *Acanthamoeba* trophozoites (**Figure 3C**). Treatment with 300, 400 and 500 μM AZ9482 led to significant reductions in trophozoites viability by 26.58% ± 4.92%, 25.96% ± 7.66% and 30.30% ± 8.61% after 24 h. More significant reductions were observed at 300, 400 and 500 μM AZ9482 after 48 h (20.90% ± 6.24%, 32.53% ± 5.30%, and 57.57% ± 4.74%) and 72 h (37.01% ± 5.44%, 49.34% ± 4.06%, and 62.58% ± 1.90%), respectively, in each group compared with the control. Simultaneously, observation by CFSE staining showed that trophozoites treated with 0.2% DMSO for 24 h had a normal shape characterized by well-spread and extended cellular morphology with distinct cytoplasmic features (**Supplementary Figure S3A**). Nevertheless, trophozoites progressively became rounded and elliptical with intracellular vacuoles gradually disappeared and finally clustered together after 300 and 400 μM AZ9482 treatment (**Supplementary Figures S3B, C**). Collectively, these *in vitro* experiments demonstrate the inhibitory effect of AZ9482 on *A. castellanii* trophozoite growth.

### 3.4 Inhibitory compounds cause trophozoite necrotic cell death rather than apoptosis

**Figure 4:**
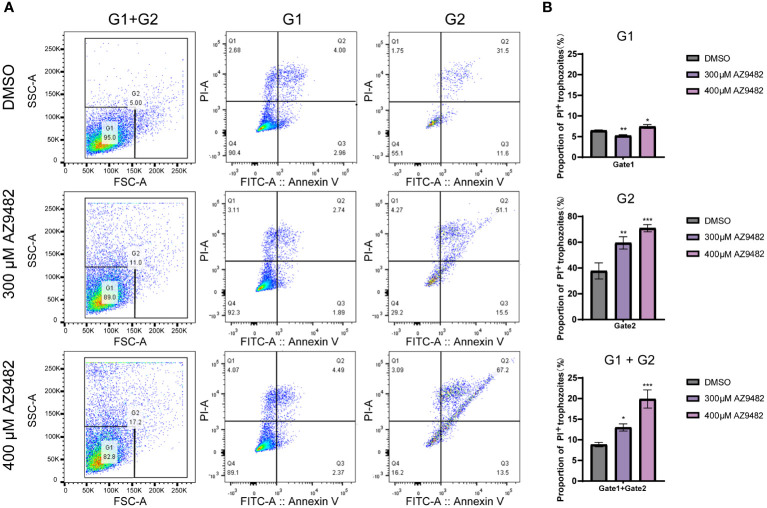
Effects of apoptosis and necrosis in *A*. *castellanii* trophozoites through 300 and 400 μM AZ9482 treatments. **(A)** Trophozoites were treated with the indicated compound concentrations for 24 h. Apoptosis rate and necrosis was measured using Annexin V-FITC/PI flow cytometry. G1: gate1 decided by 95% normal trophozoites of control group; G2: gate 2 contained the remained 5% trophozoites of control group. **(B)** Percentage of PI positive trophozoites in each gate. Data represent means ± SDs of three separate experiments (*p < 0.05, **p < 0.01, ***p < 0.001).

To investigate whether AZ9482 induced inhibition of trophozoite viability is linked to apoptosis, we evaluated phosphatidylserine levels on apoptotic trophozoite membranes using Annexin V-FITC/PI apoptosis detection. After the trophozoites treated with AZ9482, there were significant changes in the size and/or cell morphology of trophozoites. Base on size and morphology difference, the detected trophozoites were divided into two populations: G1 (gate decided by 95% normal trophozoites of control group) and G2 (gate contained the remained 5% trophozoites of control group). As depicted in **Figure 4A**, a higher percentage of necrosis were detected after 24 h of AZ9482 exposure and the percentage of early apoptotic trophozoites exhibited no significant difference. Among G1, the percentage of PI positive trophozoites showed a significantly greater increase in the 400 μM AZ9482 group (8.35% ± 0.51%) compared with the control group (6.9% ± 0.21%), whereas the 300 μM AZ9482 group (6.05% ± 0.24%) exhibited a slight decrease (**Figure 4B**). Among G2, both 300 μM AZ9482 group (60.75% ± 4.72%) and 400 μM AZ9482 (68.36% ± 6.22%) group exhibited a significantly greater increase compared with the control group (35.68% ± 5.65%) (**Figure 4B**). Obviously, a notable increase in necrosis levels was observed both in the 300 μM AZ9482 group (13.01% ± 0.86%) and 400 μM AZ9482 group (19.91% ± 2.21%) compared with the control group (8.85% ± 0.54%) (**Figure 4B**).

### 3.5 Transcriptomic landscape of *A. castellanii* trophozoites treated with AZ9482

**Figure 5:**
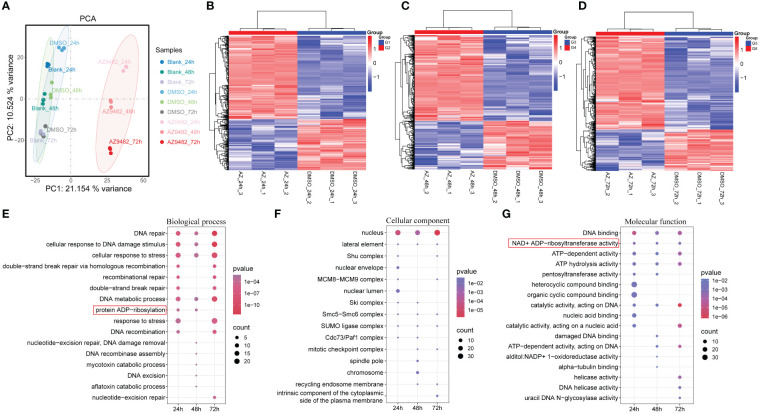
Transcriptomics analysis of trophozoites after 300 μM AZ9482 treatment for 24 h, 48 h and 72 h. **(A)** Principal-component analysis of differential groups of trophozoites by using all differentially expressed genes (n = 3). Blank: normal-cultured trophozoites. DMSO: 0.15% DMSO-treated trophozoites. AZ9482: 300 μM AZ9482-treated trophozoites. **(B–D)** Heat map of differentially expressed genes in trophozoites under 300 μM AZ9482 treatment for 24 h **(B)**, 48 h **(C)**, and 72 h **(D)**. **(E–G)** Gene Ontology (GO) functional enrichment analysis of the differentially upregulated genes. Biological process **(E)**, cell component **(F)**, molecular function **(G)**.

We first investigated the transcript profile of *A. castellanii* trophozoites following a 24 h, 48 h and 72 h treatments of AZ9482 at 300 μM concentration, aiming to capture the initial transcriptional changes induced by this small molecule. RNA-seq analysis was conducted on three sets of parallel samples: normal-cultured, DMSO-treated, and AZ9482-treated groups. Principal-component analysis (PCA) confirmed the consistency of RNA-seq results across three independent replicates (**Figure 5A**). Heat maps were shown in **Figures 5B–D**, in which the red and blue regions represented upregulated and downregulated genes with significant changes in differential abundance [log2FC < −1 or log2FC > 1; *p* < 0.05 based on Student’s t-test with false discovery rate (FDR) correction]. Specifically, at 24 h post-treatment (**Figure 5B**), 413 genes showed significant changes, with 256 upregulated and 157 downregulated. At 48 h (**Figure 5C**), 382 genes were altered, with 244 upregulated and 138 downregulated. At 72 h (**Figure 5D**), 482 genes exhibited significant changes, 333 upregulated and 149 downregulated. To categorize the differentially expressed genes (DEGs), Gene Ontology (GO) term enrichment for significantly upregulated genes were performed to three fundamental groups: biological process (BP), cellular component (CC), and molecular function (MF) (**Figures 5E–G**). Unsurprisingly, many processes related to DNA damage repair were enriched in the upregulated modules. BP analysis (**Figure 5E**) indicated predominant involvement in DNA repair, cellular response to DNA damage stimulus, cellular response to DNA damage stimulus, stress response, and various DNA metabolic process. In the CC category (**Figure 5F**), upregulated DEGs were associated mainly with nuclear components, while MF analysis showed enrichment in DNA binding, NAD^+^ ADP-ribosyltransferase activity, ATP-dependent activity and ATP hydrolysis activity. PARPs are demonstrated to promote ADP-ribosylation and their catalytic domains transfer the ADP-ribose moiety from NAD^+^ to amino acid residues of target proteins, leading to mono or poly-ADP-ribosylation (MARylation or PARylation), regulating various key biological and pathological processes (Fehr et al., 2020). This transcriptomic response discrepancy of trophozoites underscores the biological processes to counter stress during AZ92482 treatment.

### 3.6 Inhibitory compounds alter mRNA expression and induces DNA damage in trophozoites

**Figure 6:**
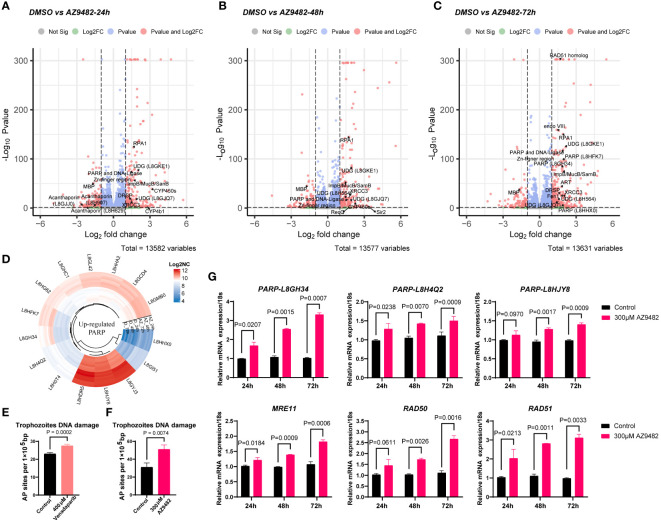
Gene expression profiles and DNA damage quantification in trophozoites treated with AZ9482. **(A–C)** Enhanced volcano plot of the differentially expressed genes (DEGs) in trophozoites under 300 μM AZ9482 treatment for 24, 48, and 72 h. The DNA damage repair pathway and virulence genes found to be statistically significant (|Fold change| >=1.5, Pvalue <=0.05) are annotated. *PARP*: Poly [ADP-ribose] polymerase. *RPA1*: Replication factora protein 1 (Rpa1) subfamily protein. *UDG*: UracilDNA glycosylase. *DRSP*: DNA repair system protein. *CYP450s*: Cytochrome p450 superfamily protein. *MBP*: mannose-binding protein (L8GXW7). *ART*: NAD(+) ADP-ribosyltransferase. *Fen1*: Flap endonuclease 1. **(D)** Circular heatmap of upregulated PARP genes in *A. castellanii* trophozoites under 300 μM AZ9482 treatment for 24h. Log2NC: log2 (normalized count). **(E, F)** Accumulation of AP sites in trophozoites with venadaparib and AZ9482 treatment for 24 h, with DNA damage assessed using commercially available assays. Differences were analyzed using the unpaired two- tailed t-test. **(G)** Relative mRNA expression of *PARP* (L8GH34), *PARP* (L8H4Q2), *PARP* (L8HJY8), *RAD51*, *RAD50*, and *MRE11* under 300 μM AZ9482 treatment for 24, 48, and 72 h in *A. castellanii* trophozoites. AZ9482 significantly promotes *PARP* (L8GH34), *PARP* (L8H4Q2), *PARP* (L8HJY8), *RAD51*, *RAD50*, and *MRE11* mRNA expression. Gene expression was normalized to 18s expression levels. Results represent means ± SDs of three independent experiments.

Our investigation into the transcript profile of *Acanthamoeba castellanii* trophozoites post-treatment with AZ9482 at 300 μM for 24, 48, and 72 h revealed notable findings. The subsequent volcano plot illustrated several DNA damage repair genes, including *RPA1* (L8GZQ4), *UDG* (L8GKE1 and L8HFK7), *ImpB/MucB/SamB* (L8GTM2), *XRCC3* (L8GYS6), and *DRSP* (L8H9W1), upregulated across all time points (24 h, 48 h, and 72 h) (**Figures 6A, B**). Furthermore, additional DNA damage repair genes exhibited significant upregulation after 72 h of AZ9482 treatment, such as *PARP* (L8GH34 and L8HFK7), *RAD51 homolog* (L8H3I3), *endo VIII* (L8GV88), *ART* (L8GZ26), *Fen1* (L8GVR2), and *UDG* (L8H564) (**Figure 6C**). Circular heatmap obviously represented 15 PARPs significantly upregulated during AZ9482 treatment (**Figure 6D**). Interestingly, upon further analysis of down-regulated genes following the 24 h treatment, we found the following virulence-related genes to be altered in trophozoites: *acanthaporin* (L8GJJ0), *acanthaporin* (L8H629), *acanthaporin* (L8H907) and *MBP* (L8GXW7, mannose-binding protein) (**Figure 6A**). Acanthaporin, to our knowledge the first well-known virulence factor to be described from *Acanthamoeba*, is considered essential for host tissue destruction (Michalek et al., 2013). Meanwhile, among these genes, only *MBP* still showed downregulated following 48 h and 72 h treatment (**Figures 6B, C**). In silico alignment, the *MBP* (L8GXW7) shares 19.4% identity (94 similar positions) with *MBP1* (Q6J288) (de Souza Gonçalves et al., 2019), which was first identified as a 400 KDa surface protein composed by multiple 130 KDa subunits (Garate et al., 2004). The mannose-binding protein (MBP) of *Acanthamoeba* is thought to play a key role in the pathogenesis of the infection by mediating the adhesion of parasites to the host cells (Hurt et al., 2003; Garate et al., 2004). Overall, these experiments indicate that AZ9482 rapidly upregulates a multitude of DNA damage repair pathways in *A. castellanii* trophozoites, and intriguingly downregulates several virulent genes. In this scenario, we next measured the rate of apurinic/apyrimidinic (AP) sites in DNA (per 10^5^ base pairs) increased from 23.0 (control group) to 27.5 (400 μM venadaparib group) and to 50.7 (300 μM AZ9482 group), further verifying probable PARPi-induced DNA damage (**Figures 6E, F**). Further validation through RT-PCR confirmed increased expression of DNA damage repair genes, including *PARP* (L8GH34, L8H4Q2 and L8HJY8), *RAD51*, *RAD50*, and *MRE11*, upon AZ9482 treatment across all time points (**Figure 6G**). These findings highlight the impact of AZ9482 on gene expression profiles, particularly in relation to protein ADP-ribosylation and DNA damage repair, ultimately inducing DNA damage in trophozoites.

## 4 Discussion

*Acanthamoeba* spp. are responsible for severe human infections, posing significant challenges for effective and targeted therapies. Current treatments lack both efficiency and specificity, potentially leading to corneal damage due to their cytotoxic nature (Fanselow et al., 2021). Additionally, the ability of *Acanthamoeba* to transition between the infective trophozoite stage and dormant cyst stage, coupled with its resilience in harsh environments, further complicates drug development (Siddiqui and Khan, 2012). These unique pathogenic features cause barriers in identifying suitable drug targets that not only directly impact the disease but also yield statistically significant therapeutic effects upon treatment. Although progress has been made in identifying novel molecular targets for *Acanthamoeba* infections over recent decades (Elsheikha et al., 2020), with various targets/functions in *Acanthamoeba* being affected by antiseptics [such as acriflavine and proflavine (Nagington and Richards, 1976; Rice et al., 2020)], antibiotics (including polymyxin B and E), and antifungals [e.g., amphotericin B (Iqbal et al., 2020)], the need for validated drug targets that address the disease’s challenges remains unmet. Therefore, identification of new anti-*Acanthamoeba* drugs with distinct mechanisms of action and efficacy against both stages is urgently needed.

PARPs constitute a family of nuclear and cytoplasmic proteins that post-translationally modify target proteins by conjugating polymeric chains of ADP-ribose during a variety of biological processes. They play fundamental roles in a number of cellular processes, including DNA repair, genomic stability, and apoptosis. Among them, PARP1 is a cellular stress sensor activated by oxidative, metabolic, and genotoxic stresses, such as single-strand break repair and double-strand DNA breaks. In response, cells are directed to specific fates according to the type and strength of stress stimuli (Luo and Kraus, 2012). The functional inhibition of PARPs by PARP inhibitors is achieved through occupancy of the catalytically active site originally inhabited by nicotinamide (NAD^+^), leading to synthetic lethality in the treatment of individuals with cancer, DNA breaks, and defective homologous recombination, such as *BRCA* gene mutation (Lord and Ashworth, 2017; Ashworth and Lord, 2018). Notably, multiple PARP-targeted compounds, including olaparib, rucaparib, niraparib, and talazoparib (Kim and Nam, 2022), have been approved for cancer treatment. Exploring the cellular response of *Acanthamoeb*a PARP inhibition in a drug-repurposing manner (Ashburn and Thor, 2004; Miró-Canturri et al., 2019) is expected to provide novel insights into therapy development, especially as short-term strategies (i.e., old drug, new tricks) reduce development time and expense but provide feasible clinical solutions. Several known drugs have been found to reduce *Acanthamoeba* growth (73% and 46% inhibition under 100 µM corifungin and tigecycline treatments, respectively) (Debnath et al., 2014; Jha et al., 2014; Jha et al., 2015), indicating the potential of drug repurposing.

In the present study, we conducted bioinformatics analyses to identify and classify 28 PARPs in *A. castellanii*. Subsequently, virtual screening of approved drugs was employed to identify potential candidates with high binding affinity for a representative *A. castellanii* PARP (UniProt accession code: L8GH34). Experimental validation demonstrated the statistically significant dose- and time-dependent inhibition of trophozoites viability with AZ9482 treatment, whereas olaparib showed no inhibitory capacity. Olaparib, a classical and effective PARP1/PARP2 inhibitor (with IC_50_ values of 5 and 1 nM, respectively), is used to treat advanced ovarian cancer in individuals with germline BRCA1/2 mutations (Yang et al., 2017; Karakashev et al., 2020). Venadaparib, a potent PARP1/PARP2 inhibitor (with IC_50_ values of 1.4 and 1.0 nM, respectively), offers broader safety margins than olaparib, displaying favorable physicochemical properties, and superior anticancer effects in homologous recombination–deficient *in vitro* and *in vivo* models (Lee et al., 2023). Despite their high inhibitory efficiency in human cancer cells, olaparib and venadaparib displayed notably weaker effects against *A. castellanii*. Several factors contribute to this disparity: (1) olaparib and venadaparib specifically inhibit PARP1/PARP2 rather than all human PARPs (Menear et al., 2008; Lee et al., 2023), (2) notable amino acid variation in the ligand-binding pocket and overall structure differences between *A. castellanii* and human PARPs, and (3) the complexity and alternative pathways of *A. castellanii* likely enhance the pathogen’s survival ability. Our results suggest that AZ9482, at high concentration, exhibited a certain inhibitory effect. AZ9482 is a triple PARPs 1/2/6 inhibitor, with IC_50_ values of 1 nM, 1 nM and 640 nM for PARP1, PARP2 and PARP6, respectively (Howard et al., 2020). As for toxicity, the cell viability assay (MTS) for MDA-MB-468 cells treated with varying concentrations of the tested compound indicated for 3 days was performed to obtain an EC_50_ of 24 nM for AZ9482, while the following medical chemistry optimization at the pharmacokinetic properties resulted in two analogues named AZ0108 (cytotoxicity EC_50 _= 140 nM), and PARPYnD (cytotoxicity EC_50_ = 300 nM) (Johannes et al., 2015; Howard et al., 2020). Surprisingly, AZ9482 cause trophozoite necrotic cell death rather than apoptosis. When the trophozoites treatment by 300 μM and 400 μM AZ9482, after such genotoxic stimuli, the trophozoites were unable to cope with DNA damage response, and launched the cell death pathway. These data presented here show the unveiling of the role of in different PARP inhibitory compounds metabolism in *Acanthamoeba* trophozoite and it will important to explore genotoxic stress signaling pathway in *Acanthamoeba*. Besides, future drug optimization of AZ9482 that selectively interacted with *Acanthamoeba* PARPs rather than human PARPs is highly pursued to achieve improved therapy efficiency with reduced side-effects or toxicity.

To further capture the initial transcriptional changes and delineate the intricate regulatory systems governing genes expression levels induced by this small molecule, we first investigated the transcript profile of *A. castellanii* trophozoites following a 24 h, 48 h and 72 h treatment of AZ9482. Biological process analysis indicated that upregulated DEGs were predominantly involved in DNA repair, recombination repair, DNA metabolic process, and protein ADP-ribosylation. Circular heatmap obviously represented 15 PARPs significantly upregulated during AZ9482 treatment, including the representative *A. castellanii* PARPs (L8GH34, L8H4Q2 and L8HJY8) (Perina et al., 2014). Notably, exposure to AZ9482 led to an increase in AP or abasic sites, prevalent lesions resulting from oxidative DNA damage. Consistent with the transcriptomic analyses, our mRNA expression data revealed upregulation of several vital DNA damage-related genes, such as *MRE11*, *RAD50* and *RAD51*. Double-strand DNA breaks (DSB) can be recognized by the MRE11-RAD50-NBS1 (MRN) complex and RAD50 containing ATPase domains interacts with MRE11 and associates with the DNA ends of the DSB (Ciccia and Elledge, 2010). RAD51 is a critical protein that facilitates the invasion of the complementary DNA strand, which is essential for the resynthesis of damaged DNA sequences. Taken together, these results, coupled with the transcriptic DEGs known to be involved in DNA damage repair, suggest that the compound AZ9482 induces DNA replication stress and activate DNA damage repair in a coordinated manner. DNA damage repair involves intricate signal transduction pathways employing an array of enzymatic tools to sense replication stress and transmit information, influencing cellular responses to mitigate the deleterious effects of aberrant DNA structures. Given that PARP inhibitors impact cancer cells without BRCA deficiency, and *Acanthamoeba* is a single-cell organism with a normal BRCA, this mechanism could potentially play a vital role in drug action. During the further analysis of transcriptomic data, we simultaneously observed a significant decrease in the expression of virulence genes after AZ9482 treatment at 24 h, such as *acanthaporin* (L8GJJ0), *acanthaporin* (L8H629), *acanthaporin* (L8H907) and *MBP* (L8GXW7). Particularly noteworthy was the significant decrease in the expression of *MBP* at 24 h, 48 h, and 72 h post-treatment, indicating a potential impact of PARP inhibitor on the inherent virulence of *A. castellanii* trophozoites. Further investigation is warranted to elucidate this phenomenon.

In summary, our study provides novel insights into the modulatory effects of PARP inhibitors on *A. castellanii* across three distinct levels: bioinformatics, cellular responses, and representative gene expression. The innate ability of *Acanthamoeba* to withstand harsh environmental conditions necessitates the development of combinational therapies that target both trophozoite and cyst stages. Additionally, drug modification, and optimization directed at *Acanthamoeba* targets are warranted. Considering the unique biological characteristics of *Acanthamoeba*, it is imperative to explore and identify unique enzyme and pathway inhibitors that can be leveraged for therapeutic interventions.

## Data availability statement

The original contributions presented in the study are included in the article/**Supplementary Material**. Further inquiries can be directed to the corresponding authors.

## Author contributions

LC: Writing – original draft, Methodology. WH: Methodology, Software, Writing – review & editing. WJ: Methodology, Writing – review & editing. MF: Methodology, Writing – review & editing. QZ: Funding acquisition, Software, Writing – review & editing. XC: Conceptualization, Funding acquisition, Project administration, Writing – review & editing.

## Supplementary material

Supplementary Figure 1 Sequence alignment of the ligand-binding pocket within the catalytic domain of human and *A. castellanii* PARPs. Residues within 4.5 Å of inhibitors reported in human PARP1/PARP2 structures (PDB codes: 3KJD, 4TVJ, 7AAD, and 4UND) are highlighted by solid triangles.

Supplementary Figure 2 Two-dimensional (2D) diagrams of ligand–protein interactions for five ligands: AZD2461 **(A)**, KU0058948 **(B)**, talazoparib **(C)**, bopindolol **(D)** and trovafloxacin **(E)**.

Supplementary Figure 3 Representative images of *A. castellanii* trophozoites via CFSE staining using CLSM. Trophozoites were treated with 0.2% DMSO **(A)**, 300 μM AZ9482 **(B)** or 400 μM AZ9482 **(C)** for 24h (20 × and 40 × magnification, bar = 50 μm). (20 × and 80 × objective magnification, bar = 50 μm).
