# Supplementary figures and images for: Novel anti-Acanthamoeba effects elicited by a repurposed poly (ADP-ribose) polymerase inhibitor AZ9482
Source: Front Cell Infect Microbiol. 2024 May 28;14:1414135. doi: 10.3389/fcimb.2024.1414135 (PMC11165085; doi:10.3389/fcimb.2024.1414135)

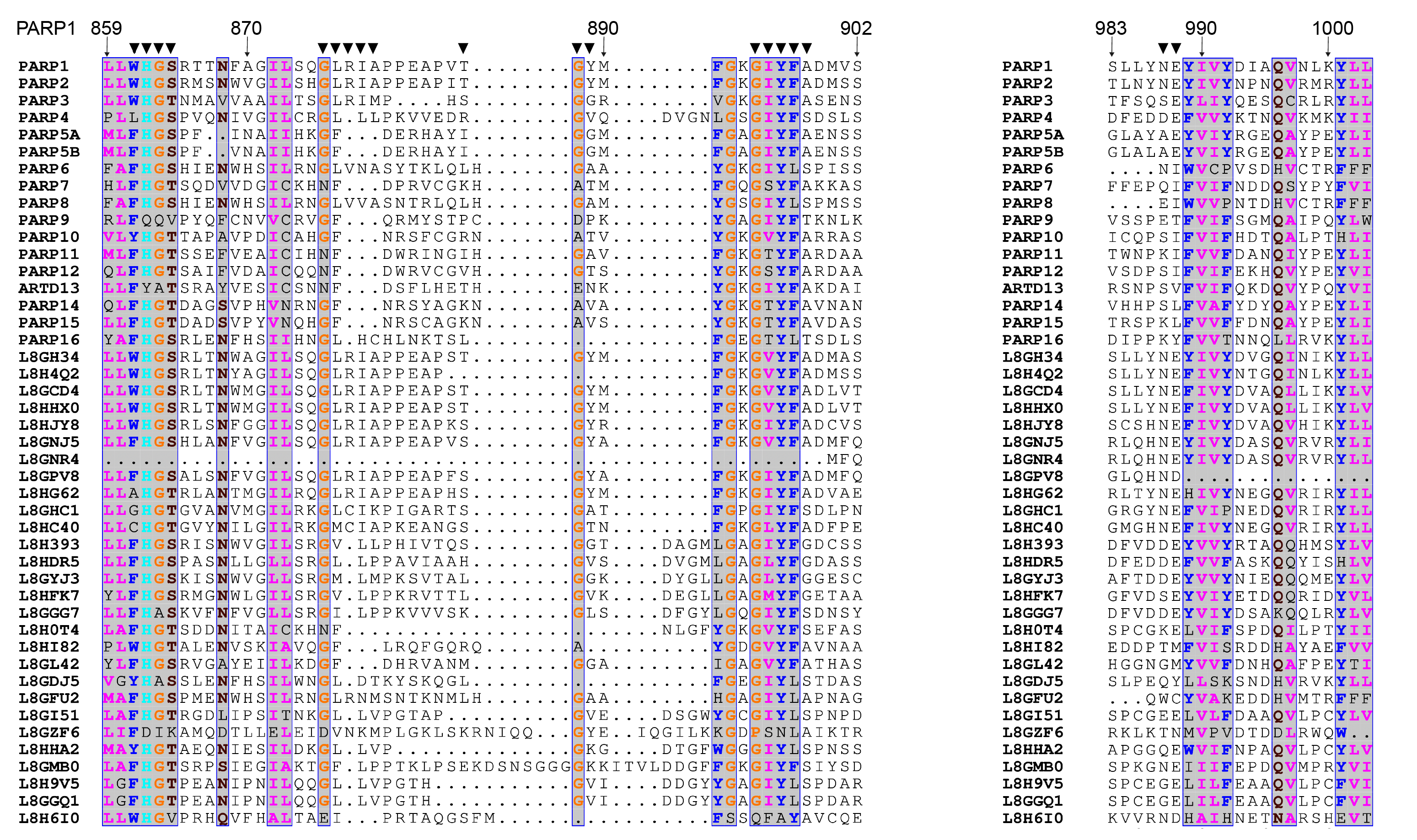

Supplement: Supplementary Figure 1 — Sequence alignment of the ligand-binding pocket within the catalytic domain of human and A. castellanii PARPs. Residues within 4.5 Å of inhibitors reported in human PARP1/PARP2 structures (PDB codes: 3KJD, 4TVJ, 7AAD, and 4UND) are highlighted by solid triangles. [file Image_1.tif]

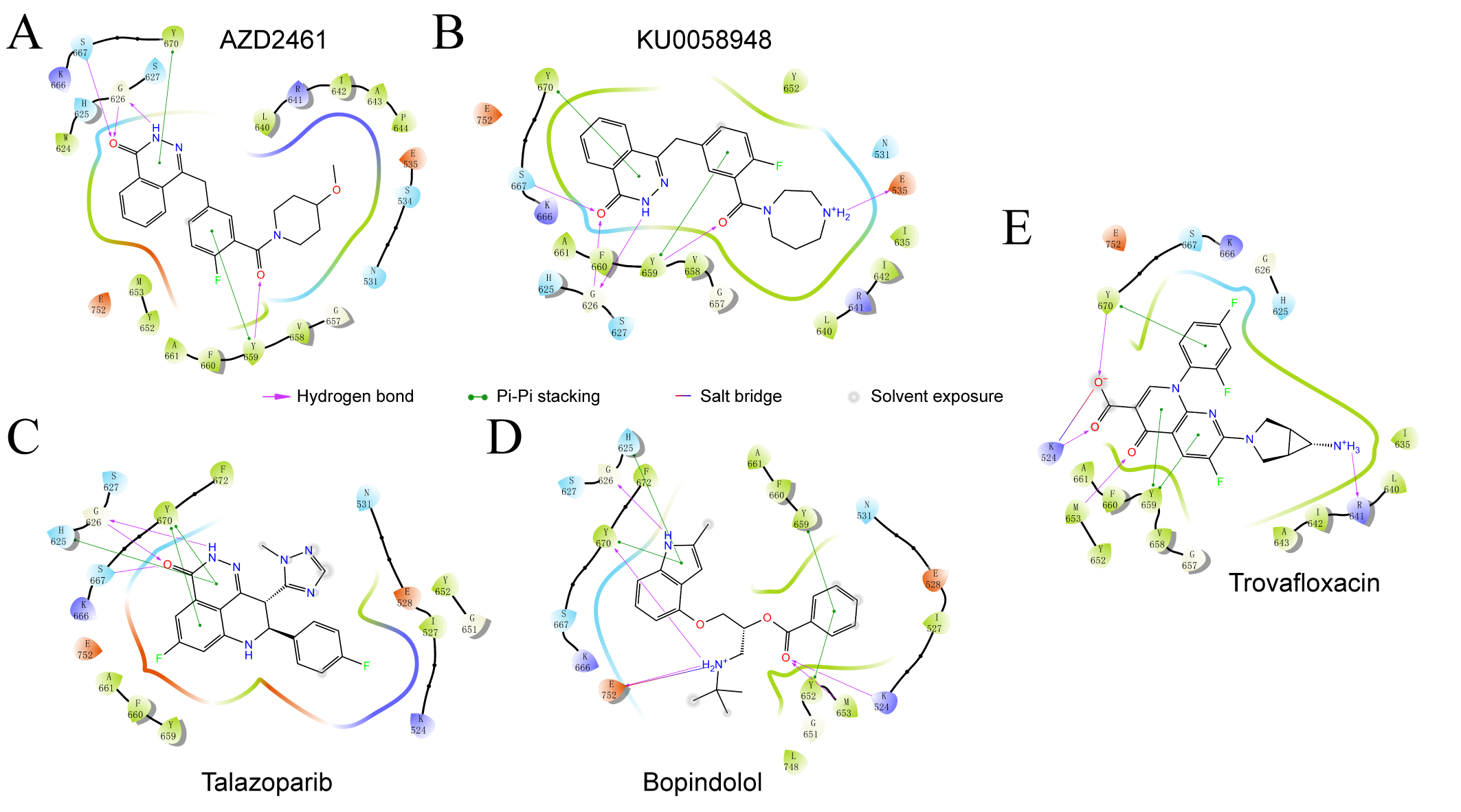

Supplement: Supplementary Figure 2 — Two-dimensional (2D) diagrams of ligand–protein interactions for five ligands: AZD2461 (A), KU0058948 (B), talazoparib (C), bopindolol (D) and trovafloxacin (E). [file Image_2.tif]

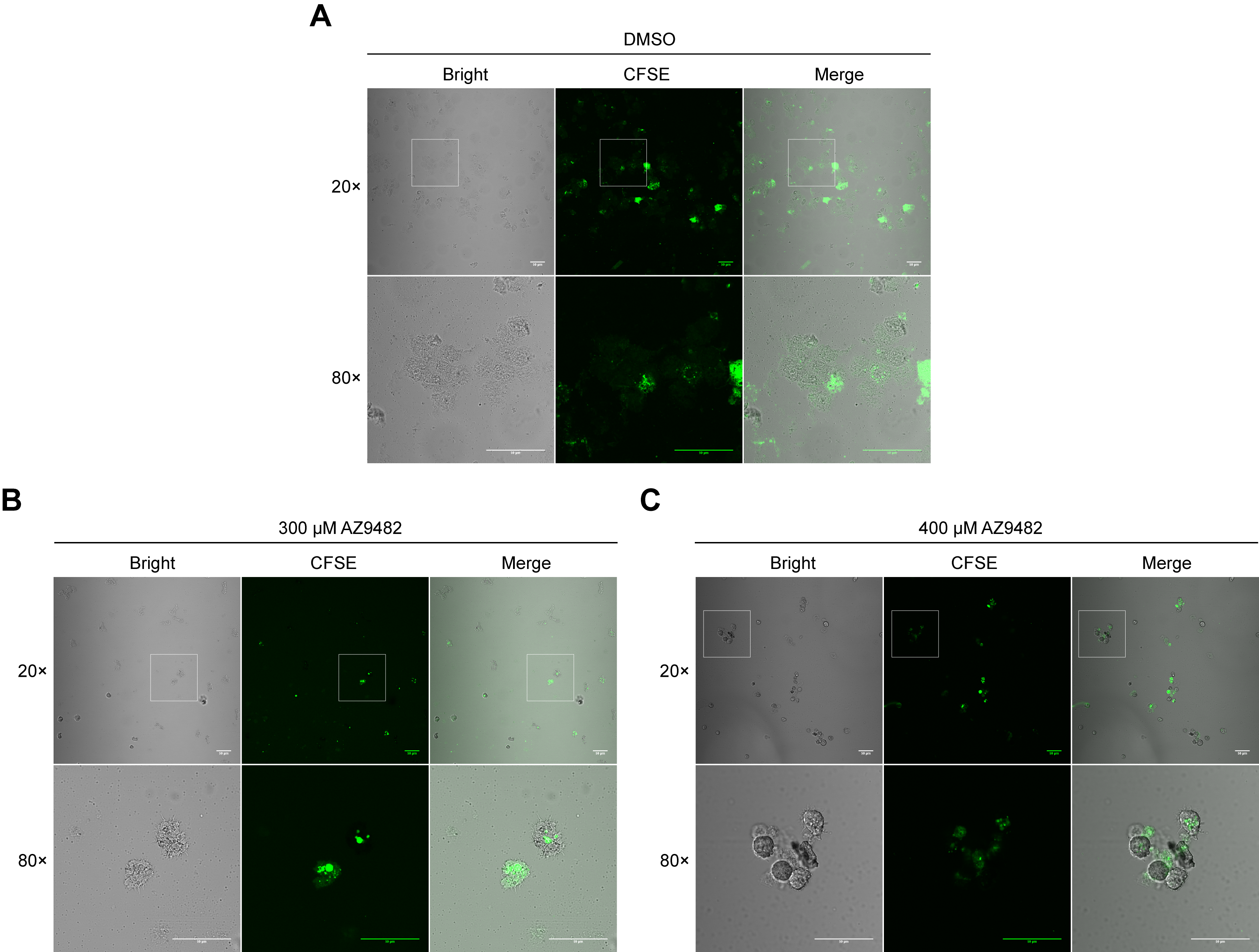

Supplement: Supplementary Figure 3 — Representative images of A. castellanii trophozoites via CFSE staining using CLSM. Trophozoites were treated with 0.2% DMSO (A), 300 μM AZ9482 (B) or 400 μM AZ9482 (C) for 24h (20 × and 40 × magnification, bar = 50 μm). (20 × and 80 × objective magnification, bar = 50 μm). [file Image_3.tif]
