# Supplementary material for: Novel anti-Acanthamoeba effects elicited by a repurposed poly (ADP-ribose) polymerase inhibitor AZ9482
Source: Front Cell Infect Microbiol. 2024 May 28;14:1414135. doi: 10.3389/fcimb.2024.1414135 (PMC11165085; doi:10.3389/fcimb.2024.1414135)
Supplement: Supplementary file 4 [file Table_1.doc]

**Table S1. Lists of poly (ADP-ribose) polymerases (PARPs) in *Acanthamoeba castellanii str. Neff.***

| **Number** | **Entry name** | **Length of protein** | **Protein name** | **Gene names** | **NCBI Reference sequence** |
| --- | --- | --- | --- | --- | --- |
| 1 | L8GCD4_ACACA | 631 | Poly (ADP-ribose) polymerase | ACA1_ 181230 | XP_004332883.1 |
| 2 | L8GGG7_ACACA | 1501 | Poly (ADP-ribose) polymerase | ACA1_ 140610 | XP_004334168.1 |
| 3 | L8GH34_ACACA | 774 | Poly (ADP-ribose) polymerase | ACA1_256450 | XP_004333519.1 |
| 4 | L8GHC1_ACACA | 1893 | Poly (ADP-ribose) polymerase | ACA1_374660 | XP_004334410.1 |
| 5 | L8GL42_ACACA | 524 | Poly (ADP-ribose) polymerase | ACA1_095660 | XP_004334929.1 |
| 6 | L8GNJ5_ACACA | 3016 | Poly (ADP-ribose) polymerase | ACA1_077830 | XP_004336565.1 |
| 7 | L8GPV8_ACACA | 2562 | Poly (ADP-ribose) polymerase | ACA1_ 131220 | XP_004336154.1 |
| 8 | L8GYJ3_ACACA | 1777 | Poly (ADP-ribose) polymerase | ACA1_321600 | XP_004340383.1 |
| 9 | L8H393_ACACA | 2088 | Poly (ADP-ribose) polymerase | ACA1_262210 | XP_004341268.1 |
| 10 | L8H4Q2_ACACA | 1098 | Poly (ADP-ribose) polymerase | ACA1_200050 | XP_004341800.1 |
| 11 | L8HC40_ACACA | 609 | Poly (ADP-ribose) polymerase | ACA1_ 149970 | XP_004351584.1 |
| 12 | L8HDR5_ACACA | 2693 | Poly (ADP-ribose) polymerase | ACA1_069620 | XP_004352905.1 |
| 13 | L8HFK7_ACACA | 1326 | Poly (ADP-ribose) polymerase | ACA1_356100 | XP_004354006.1 |
| 14 | L8HG62_ACACA | 731 | Poly (ADP-ribose) polymerase | ACA1_272710 | XP_004353775.1 |
| 15 | L8HJY8_ACACA | 1093 | Poly (ADP-ribose) polymerase | ACA1_ 173240 | XP_004356596.1 |
| 16 | L8GDJ5_ACACA | 291 | Poly (ADP-ribose) polymerase catalytic domain containing protein | ACA1_ 108300 | XP_004332806.1 |
| 17 | L8GFU2_ACACA | 742 | Poly (ADP-ribose) polymerase | ACA1_261440 | XP_004333737.1 |
| 18 | L8GGQ1_ACACA | 701 | Poly(ADP-ribose) polymerase catalytic domain containing protein | ACA1_399770 | XP_004333941.1 |
| 19 | L8GI51_ACACA | 500 | Poly (ADP-ribose) polymerase | ACA1_092950 | XP_004334773.1 |
| 20 | L8GMB0_ACACA | 463 | Poly (ADP-ribose) polymerase catalytic domain containing protein | ACA1_364190 | XP_004335906.1 |
| 21 | L8GNR4_ACACA | 135 | Poly (ADP-ribose) polymerase | ACA1_077800 | XP_004336562.1 |
| 22 | L8GZF6_ACACA | 250 | Poly (ADP-ribose) polymerase, regulatory subfamily protein | ACA1_207200 | XP_004339929.1 |
| 23 | L8H0T4_ACACA | 601 | Poly (ADP-ribose) polymerase catalytic domain containing protein | ACA1_ 166970 | XP_004340895.1 |
| 24 | L8H6I0_ACACA | 405 | Poly (ADP-ribose) polymerase catalytic domain containing protein | ACA1_ 114300 | XP_004342171.1 |
| 25 | L8H9V5_ACACA | 363 | Poly (ADP-ribose) polymerase catalytic domain containing protein | ACA1_327900 | XP_004346047.1 |
| 26 | L8HHA2_ACACA | 1150 | Poly (ADP-ribose) polymerase | ACA1_301550 | XP_004356983.1 |
| 27 | L8HHX0_ACACA | 216 | Poly (ADP-ribose) polymerase | ACA1_338640 | XP_004354000.1 |
| 28 | L8HI82_ACACA | 729 | Poly (ADP-ribose) polymerase | ACA1_ 176050 | XP_004356827.1 |
