# Supplementary material for: Novel anti-Acanthamoeba effects elicited by a repurposed poly (ADP-ribose) polymerase inhibitor AZ9482
Source: Front Cell Infect Microbiol. 2024 May 28;14:1414135. doi: 10.3389/fcimb.2024.1414135 (PMC11165085; doi:10.3389/fcimb.2024.1414135)
Supplement: Supplementary file 5 [file Table_2.doc]

**Table S2. Primer sequences used for real-time PCR**

| Name | Primer | Sequence（5′–3′） | Reference |
| --- | --- | --- | --- |
| 18s rDNA | Forward  Reverse | TCCAATTTTCTGCCACCGAA  ATCATTACCCTAGTCCTCGCGC |  |
| PARP-L8GH34 | Forward  Reverse | GTTGTGGGCCAACTACCAGA  AGTTGGTCAATCGCGAACCA | XM_004333471.1 |
| PARP-L8H4Q2 | Forward  Reverse | TCCAAGTACGTGACGCAGAC  GCTGCTCATGTCCGCAAAAT | XM_004341752.1 |
| PARP-L8HJY8 | Forward  Reverse | CGTGACGGCATCTCCAAGAA  CTGACGGTAGAGGCTCTGGT | XM_004356543.1 |
| RAD50 | Forward  Reverse | AAGGAGGAGAACGTGGCAAG  CTTGTTTTCGTTGGCTCGCA | XM_004339591.1 |
| RAD51 | Forward  Reverse | GCGGACCCATTCCTATCACC  TGGTCGAAAAAGCCACCGAT | XM_004341844.1 |
| MRE11 | Forward  Reverse | CTTCTCCGTCGCTACTGCAT  AAGGATCGGGTAGACCGTGA | XM_004339616.1 |
